# Pan-Cancer Analysis Identifies SNORA12 as a Prognostic Biomarker and Demonstrates Its Role in Upregulating TIGIT in Osteosarcoma

**DOI:** 10.3390/biomedicines14030723

**Published:** 2026-03-20

**Authors:** Weiwei He, Wenbo Shi, Qian Li, Baiguang Yu, Jia Song, Marina Igorevna Sekacheva, Haiyan Hu

**Affiliations:** 1 Clinical Research Ward, The Sixth People’s Hospital of Shanghai Jiao Tong University School of Medicine, Shanghai 200233, China; hww18930172450@163.com (W.H.); 13860644676@163.com (W.S.); liqian990727@163.com (Q.L.); ybg2016@126.com (B.Y.); songjialc@163.com (J.S.); 2 Department of Thoracic Surgery, The Sixth People’s Hospital of Shanghai Jiao Tong University School of Medicine, Shanghai 200235, China; 3 Department of Innovative Healthcare Development, Institute of Personalized Oncology, Sechenov University, 119991 Moscow, Russia

**Keywords:** SNORA12, osteosarcoma, biomarker

## Abstract

**Background:** Small nucleolar RNAs (snoRNAs) are emerging regulators of tumorigenesis, yet their pan-cancer landscape and immunological roles remain poorly defined. This study investigates the expression pattern, prognostic significance, and immune correlation of SNORA12 across cancers, with mechanistic validation in osteosarcoma. **Methods:** We integrated RNA-seq data from the TCGA, TARGET, and GTEx databases to evaluate SNORA12 expression and its prognostic value using Cox regression and Kaplan–Meier analyses (progression-free survival, PFS). The correlation between SNORA12 and the tumor immune microenvironment was assessed using six independent algorithms (TIMER, EPIC, CIBERSORT, IPS, MCP-counter, xCELL). In vitro, the regulatory effect of SNORA12 on the immune checkpoint TIGIT was validated by overexpression and knockdown experiments in osteosarcoma cell lines (SW1353, U2OS) and NK cells. **Results:** SNORA12 expression exhibited significant tumor-type specificity. High SNORA12 expression was associated with poor prognosis in glioma (HR = 1.31, *p* = 0.006) but favorable outcomes in pancreatic (HR = 0.51, *p* = 0.01) and breast cancer (HR = 0.56, *p* = 0.02). Immunologically, SNORA12 showed robust positive correlations with CD8+ T cell infiltration in thyroid carcinoma (THCA) and lung adenocarcinoma (LUAD) across multiple algorithms. Notably, SNORA12 expression was positively correlated with m6A modifiers METTL3 and YTHDF1, and negatively correlated with the demethylase FTO. Experimentally, overexpression of SNORA12 in osteosarcoma cells and primary NK cells significantly upregulated TIGIT at both the mRNA and protein levels, while SNORA12 knockdown in NK92 cells reduced TIGIT expression. **Conclusions:** This pan-cancer analysis positions SNORA12 as a tumor type-specific prognostic biomarker and reveals its novel role as a positive regulator of TIGIT in osteosarcoma, offering a potential mechanistic link between snoRNA dysregulation and immune evasion.

## 1. Introduction

Cancer remains a leading cause of mortality worldwide, posing a significant public health challenge with an estimated 19.29 million new cases and 9.96 million deaths in 2020 [1].

Beyond protein-coding genes, non-coding RNAs (ncRNAs) have emerged as critical regulators of cancer biology. This class includes microRNAs (miRNAs), long non-coding RNAs (lncRNAs), and small nucleolar RNAs (snoRNAs) [2,3]. While the roles of miRNAs and lncRNAs in tumor proliferation and metastasis have been extensively characterized [2,3], the function of snoRNAs in cancer, particularly in immune modulation, is less understood.

SnoRNAs are predominantly nucleolar RNAs traditionally known for guiding chemical modifications—such as 2′-O-ribose methylation and pseudouridylation—of ribosomal RNA (rRNA), thereby influencing ribosome biogenesis and function [4]. SNORA12, a member of the box H/ACA snoRNA family, possesses conserved H (ANANNA) and ACA motifs [4] and is predicted to guide rRNA pseudouridylation. Recent evidence has hinted at the dysregulation of snoRNAs in malignancy; for instance, high SNORA12 expression has been linked to breast cancer progression and chemoresistance [4]. However, its expression landscape, prognostic value, and potential involvement in the tumor immune microenvironment (TIME) across diverse cancer types remain largely unexplored.

The immune checkpoint TIGIT (T cell immunoreceptor with Ig and ITIM domains) has emerged as a promising therapeutic target, yet the regulatory networks controlling its expression—particularly those involving non-coding RNAs—remain poorly defined. In this study, we performed a comprehensive pan-cancer analysis to elucidate the expression patterns and clinical significance of SNORA12. We identified significant correlations between SNORA12 expression and immune cell infiltration, especially with key RNA modification enzymes. While our pan-cancer analysis revealed heterogeneous SNORA12 expression across multiple tumor types, we selected osteosarcoma for mechanistic validation based on our preliminary findings: single-cell sequencing of osteosarcoma samples unexpectedly revealed high infiltration of TIGIT+ Treg cells (>10% in some patients), and functional screening of patient-derived exosome contents identified SNORA12 among five highly abundant ncRNAs as a candidate regulator of TIGIT expression, associated with an 11.48% upregulation and concurrent MEK/ERK pathway activation. These observations provided a unique opportunity to explore a potential SNORA12-TIGIT axis in a clinically relevant context. Our findings demonstrate that SNORA12 acts as a positive regulator of TIGIT in both osteosarcoma cells and NK cells, suggesting a potential mechanism for snoRNA-mediated immune evasion.

## 2. Materials and Methods

### 2.1. Study Design and Data Acquisition

This study aimed to systematically evaluate the pan-cancer expression pattern, prognostic significance, and immune association of SNORA12, and to preliminarily explore its potential role in natural killer (NK) cell biology. To achieve this, we employed an integrated strategy combining large-scale bioinformatics analysis with subsequent molecular and cellular validation.

#### 2.1.1. Multi-Cohort Gene Expression Data Processing

RNA sequencing data (in transcripts per million, TPM) and corresponding clinical information for 33 cancer types were retrieved from The Cancer Genome Atlas (TCGA) and the Genotype-Tissue Expression (GTEx) project via the UCSC Xena portal. Additional pediatric cancer data were obtained from the TARGET database. Raw expression values were standardized across all samples. To approximate a normal distribution and reduce the impact of extreme values, expression data were transformed using log2(TPM + 1). Cancer types with fewer than 10 tumor samples were excluded from subsequent analyses to ensure statistical reliability. To maximize sample coverage and leverage normal tissue controls where appropriate, the number of cancer types analyzed varied by algorithm. For analyses relying exclusively on tumor samples (e.g., TIMER, differential expression, and clinical stage correlations), we included 38 cancer types from TCGA and GTEx. For algorithms capable of integrating GTEx normal tissue data to improve stromal and immune cell scoring accuracy (e.g., EPIC, xCELL, IPS, MCP-counter, CIBERSORT), we expanded the analysis to 44 cancer types, as previously described [5,6].

#### 2.1.2. Survival and Prognostic Analysis

The prognostic value of SNORA12 expression was assessed using progression-free survival (PFS) data. Univariate Cox proportional hazards regression models were constructed for each cancer type using the coxph function from the survival package in R (version 4.3.0). Patients within each cancer cohort were dichotomized into SNORA12 high- and low-expression groups based on the median expression value. Kaplan–Meier survival curves were generated, and differences between groups were compared using the log-rank test. Hazard ratios (HR) with 95% confidence intervals (CI) were derived from the Cox models. The proportional hazards assumption was verified using Schoenfeld residuals.

#### 2.1.3. Analysis of Immune Cell Infiltration

To comprehensively evaluate the correlation between SNORA12 expression and tumor immune microenvironment composition, we applied six independent deconvolution algorithms: TIMER, EPIC, CIBERSORT, IPS, MCP-counter, and xCELL. Each algorithm estimates the abundance of specific immune cell populations based on gene expression profiles. The association between SNORA12 expression and immune cell infiltration scores was quantified using Spearman’s rank correlation coefficient. To identify the most robust associations, results were cross-validated across multiple algorithms, and an UpSet plot was constructed to highlight cancer types with consistent correlations (Supplementary Figure S1).

### 2.2. Experimental Validation

#### 2.2.1. Cell Culture

Human NK-92 cell line (ATCC^®^ CRL-2408™, Manassas, VI, USA) was maintained in Alpha MEM medium supplemented with 12.5% fetal bovine serum (FBS), 12.5% horse serum, 0.1 mM 2-mercaptoethanol, and recombinant human IL-2 (100 U/mL). Cells were kept in a humidified incubator at 37 °C with 5% CO_2_.

#### 2.2.2. Plasmid Construction and Transfection

To investigate the functional role of SNORA12, we constructed both overexpression and knockdown plasmids. The full-length sequence of SNORA12 was synthesized and cloned into the pcDNA3.1(+) vector (Genomeditech, Shanghai, China). For knockdown experiments, short hairpin RNA (shRNA) targeting SNORA12 (sh-SNORA12) and a negative control scramble sequence (sh-NC) were cloned into the pLKO.1 vector. SW1353, U2OS, and primary NK cells were transfected with the overexpression plasmid (OE-SNORA12) or empty vector (OE-NC) using Lipofectamine 3000 (Invitrogen, Carlsbad, CA, USA) according to the manufacturer’s instructions. NK92 cells were transduced with lentivirus containing sh-SNORA12 or sh-NC. Transfection and knockdown efficiency were validated by quantitative real-time PCR (qRT-PCR) 48 h post-transfection (Supplementary Figure S2).

#### 2.2.3. Quantitative Real-Time PCR (qRT-PCR)

Total RNA was isolated from cells using TRIzol reagent (Vazyme, Nanjing, China) according to the manufacturer’s protocol. cDNA was synthesized from 1 μg of total RNA using the PrimeScript RT Reagent Kit (Vazyme). Quantitative PCR was performed using ChamQ Universal SYBR qPCR Master Mix (Vazyme) on a QuantStudio 5 Real-Time PCR System (Applied Biosystems, Waltham, MA, USA). The relative expression level of SNORA12 was normalized to the endogenous control GAPDH and calculated using the 2−ΔΔCt method. All primer sequences are listed in Table 1.

#### 2.2.4. Western Blot Analysis

To investigate the protein level of the immune checkpoint TIGIT, total cellular protein was extracted using RIPA lysis buffer containing protease inhibitors. Protein concentration was determined via BCA assay. Approximately 30 μg of protein per sample was separated by 10% SDS-polyacrylamide gel electrophoresis and subsequently transferred onto nitrocellulose membranes (Millipore, Burlington, MA, USA). After blocking with 5% non-fat milk, membranes were incubated overnight at 4 °C with primary antibodies against TIGIT (1:1000, Proteintech, Cat# 66699-1-Ig, Rosemont, IL, USA) and β-actin (1:5000, serving as loading control). Following incubation with HRP-conjugated secondary antibodies, protein bands were visualized using an enhanced chemiluminescence (ECL) detection system and quantified with ImageJ software(Version 1.53e, National Institutes of Health, Bethesda, MD, USA).

#### 2.2.5. Isolation, Activation, and Purity Assessment of Primary Human NK Cells

Umbilical cord blood (UCB) units were collected from healthy full-term deliveries with informed consent under an approved institutional review board protocol. Peripheral blood mononuclear cells (PBMCs) were isolated via density-gradient centrifugation using Ficoll-Paque PLUS (GE Healthcare, Chicago, IL, USA). For NK cell expansion and activation, isolated PBMCs were cultured in RPMI-1640 medium supplemented with 10% FBS, 1% penicillin-streptomycin, recombinant human IL-2 (1000 U/mL), and IL-15 (10 ng/mL), in the presence of an NK cell activation cocktail (Miltenyi Biotec, Bergisch Gladbach, Germany) for 5–7 days. NK cell purity was assessed by flow cytometry (BD FACS Celesta, San Francisco, CA, USA) using fluorescently labeled antibodies against CD3 (FITC) and CD56 (APC). Only cultures with a CD3^−^CD56^+^ cell population exceeding 90% were used for downstream functional assays.

### 2.3. Statistical Analysis

For bioinformatics analyses, statistical significance was defined as a false discovery rate (FDR) < 0.05 after Benjamini–Hochberg correction for multiple testing where applicable. All correlation analyses (immune infiltration, RNA modification genes, immune checkpoint genes) were subjected to FDR correction. For in vitro experiments, data are presented as mean ± standard deviation (SD) from at least three independent biological replicates. Group comparisons were performed using a two-tailed Student’s *t*-test (for two groups) or one-way ANOVA with Tukey’s post hoc test (for multiple groups). A *p*-value < 0.05 was considered statistically significant. Statistical analyses were performed using R software (version 4.3.0) and GraphPad Prism (version 9.0).

## 3. Results

### 3.1. Prognostic Significance of SNORA12 Across Cancer Types

**Figure 1:**
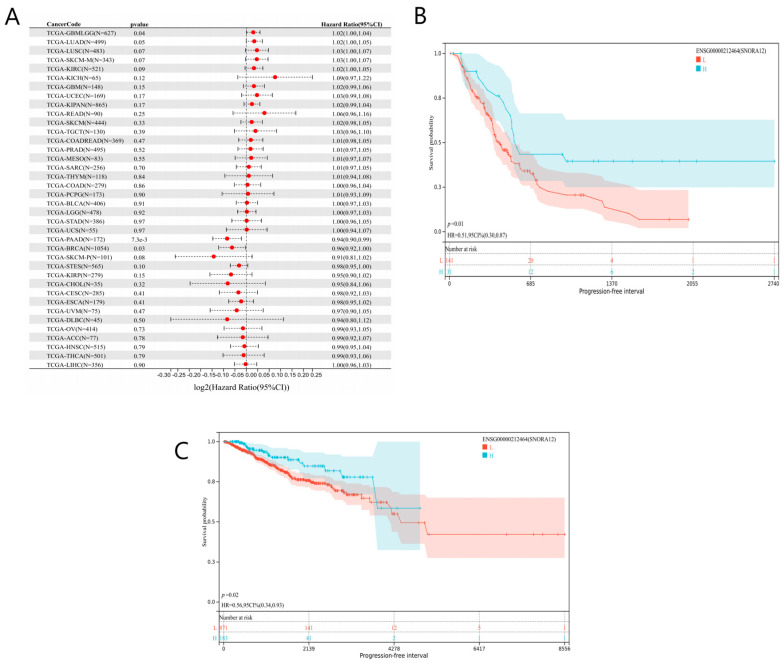
Prognostic significance of SNORA12 across cancer types. (**A**) Forest plot showing the association between SNORA12 expression and progression-free survival (PFS) in 38 cancer types from TCGA and GTEx. Hazard ratios (HR) and 95% confidence intervals (CI) were calculated using univariate Cox regression. Red indicates HR > 1 (higher expression associated with poorer prognosis); blue indicates HR < 1 (higher expression associated with better prognosis). (**B**) Kaplan–Meier survival curves comparing PFS between SNORA12 high- and low-expression groups in pancreatic adenocarcinoma (TCGA-PAAD, N = 172). Patients were stratified by median expression. *p*-value was calculated by log-rank test. (**C**) Kaplan–Meier survival curves for breast invasive carcinoma (TCGA-BRCA, N = 1077), stratified by median SNORA12 expression.

To evaluate the clinical relevance of SNORA12, we analyzed its association with progression-free survival (PFS) across 38 cancer types using univariate Cox regression. As shown in the forest plot (Figure 1A), the prognostic value of SNORA12 was highly tumor type-specific.

High SNORA12 expression was significantly associated with poor prognosis in glioma (TCGA-GBMLLGG, N = 627, HR = 1.31, 95% CI: 1.08–1.59, *p* = 0.006), suggesting a potential oncogenic role in this malignancy. Conversely, high SNORA12 expression correlated with favorable prognosis in pancreatic adenocarcinoma (TCGA-PAAD, N = 172, HR = 0.51, 95% CI: 0.30–0.87, *p* = 0.01) and breast invasive carcinoma (TCGA-BRCA, N = 1077, HR = 0.56, 95% CI: 0.34–0.93, *p* = 0.02) (Figure 1B,C). In the remaining 35 cancer types (e.g., COAD, LUAD, KIRC), the association between SNORA12 expression and PFS did not reach statistical significance (Figure 1A), further underscoring the context-dependent nature of its biological function.

These results demonstrate that SNORA12 exerts dual prognostic effects depending on tumor type, acting as a risk factor in glioma and a protective factor in pancreatic and breast cancers.

### 3.2. Correlation Between SNORA12 Expression and Immune Cell Infiltration

**Figure 2:**
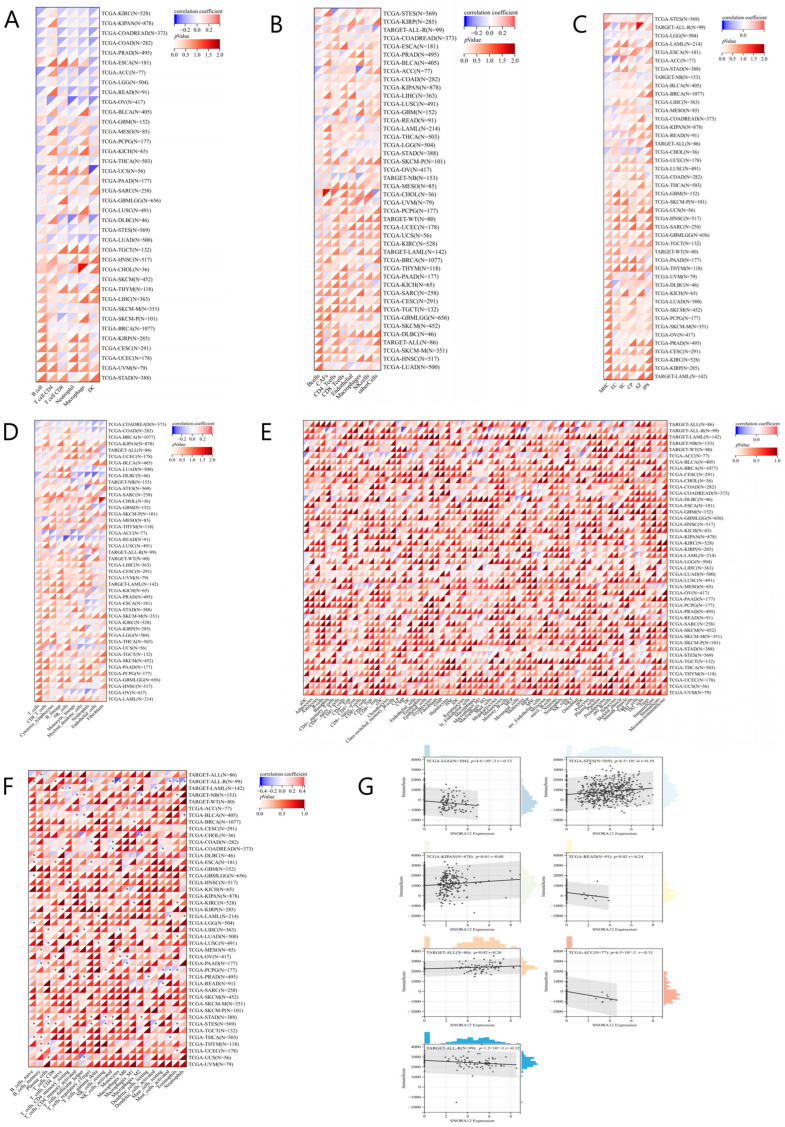
Correlation between SNORA12 expression and immune cell infiltration across cancers. Heatmaps displaying Spearman’s rank correlation coefficients (R) between SNORA12 expression and immune cell infiltration scores estimated by six independent algorithms: (**A**) TIMER (38 cancer types, 6 immune cell types), (**B**) EPIC (44 cancer types, 8 immune cell types), (**C**) IPS (44 cancer types, 6 immune cell types), (**D**) MCP-counter (44 cancer types, 8 immune cell types), (**E**) xCELL (44 cancer types, 16 immune cell types), and (**F**) CIBERSORT (44 cancer types, 22 immune cell types). Red indicates a positive correlation; blue indicates a negative correlation. The color intensity represents the magnitude of R. Asterisks denote statistical significance after FDR correction (* *p* < 0.05). (**G**) Correlation between SNORA12 expression and global immune score in 44 cancer types (N = 10,180 samples). Only cancer types with significant correlations (*p* < 0.05 after FDR correction) are labeled.

To comprehensively evaluate the relationship between SNORA12 and the tumor immune microenvironment, we employed six independent deconvolution algorithms (TIMER, EPIC, IPS, MCP-counter, xCELL, and CIBERSORT) to analyze immune cell infiltration scores across 38–44 cancer types (Figure 2A–F). In all heatmaps, red indicates a positive Spearman correlation (high SNORA12 associated with high immune infiltration), while blue indicates a negative correlation (high SNORA12 associated with low immune infiltration).

Using the TIMER algorithm (38 cancer types, N = 9406 samples), SNORA12 expression was significantly correlated with the infiltration of at least one immune cell type in 23 cancer types (Figure 2A). For example, in adrenocortical carcinoma (TCGA-ACC, N = 77), SNORA12 showed a strong negative correlation with macrophage infiltration (R = −0.41, *p* < 0.001), suggesting a potential immunosuppressive role. In breast cancer (TCGA-BRCA, N = 1077), SNORA12 was positively correlated with CD8+ T cell infiltration (R = 0.19, *p* < 0.001).

The EPIC algorithm (44 cancer types, N = 10,180 samples) revealed significant correlations in 21 cancer types (Figure 2B). Notably, in glioblastoma and low-grade glioma (TCGA-GBMLLGG, N = 656), SNORA12 expression was negatively correlated with CD8+ T cell infiltration (R = −0.21, *p* = 0.002), consistent with its poor prognostic value in this malignancy. In lung adenocarcinoma (TCGA-LUAD, N = 500), a positive correlation with NK cell infiltration was observed (R = 0.18, *p* = 0.01).

The IPS, MCP-counter, xCELL, and CIBERSORT algorithms further validated these findings, demonstrating significant correlations across 17, 31, 41, and 35 cancer types, respectively (Figure 2C–F). To identify the most robust associations, we performed a cross-algorithm comparison using an UpSet plot (Supplementary Figure S1). This analysis revealed that thyroid carcinoma (THCA) and lung adenocarcinoma (LUAD) exhibited consistent positive correlations between SNORA12 expression and CD8+ T cell infiltration across at least four independent algorithms, strongly suggesting that SNORA12 may play a conserved role in regulating adaptive immunity in these cancer types. Conversely, glioblastoma and low-grade glioma (GBMLLGG) showed consistent negative correlations in four algorithms. Additionally, seven cancer types—adrenocortical carcinoma (ACC), breast invasive carcinoma (BRCA), colon and rectal adenocarcinoma (COADREAD), esophageal carcinoma (ESCA), ovarian serous cystadenocarcinoma (OV), rectal adenocarcinoma (READ), and esophagogastric adenocarcinoma (STES)—demonstrated significant correlations across all six algorithms (Supplementary Figure S1).

### 3.3. Correlation Between SNORA12 Expression and Immune Infiltration Score

We next analyzed the association between SNORA12 expression and the global immune infiltration score across 44 cancer types (N = 10,180 samples) using Spearman correlation (Figure 2G). Significant correlations were observed in 7 cancer types after FDR correction.

Positive correlations were found in three cancer types: TCGA-STES (N = 569, R = 0.19, *p* = 6.5 × 10^−6^), TCGA-KIPAN (N = 878, R = 0.08, *p* = 0.01), and TARGET-ALL (N = 86, R = 0.26, *p* = 0.02). Negative correlations were observed in four cancer types: TCGA-LGG (N = 504, R = −0.13, *p* = 4.6 × 10^−3^), TCGA-READ (N = 91, R = −0.24, *p* = 0.02), TCGA-ACC (N = 77, R = −0.31, *p* = 6.5 × 10^−3^), and TARGET-ALL-R (N = 99, R = −0.32, *p* = 1.2 × 10^−3^). These results further support the context-dependent role of SNORA12 in shaping the immune microenvironment.

### 3.4. Correlation Between SNORA12 and RNA Modification Genes

**Figure 3:**
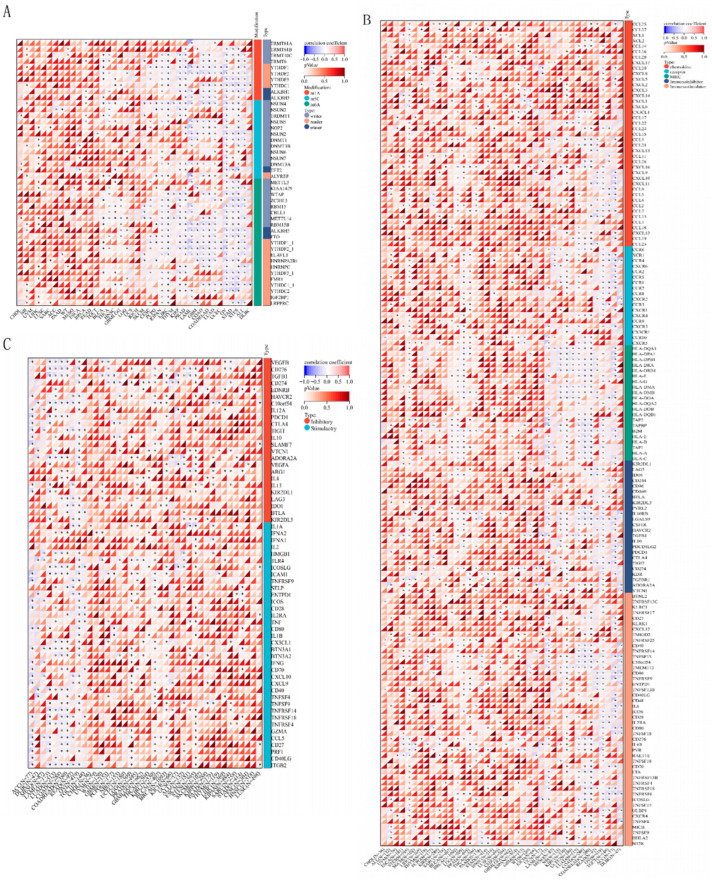
Pan-cancer correlation heatmaps between SNORA12 expression and key gene sets. (**A**) Correlation with RNA modification genes (m6A, m5C, m1A regulators). (**B**) Correlation with immune regulatory genes (chemokines, receptors, MHC molecules). (**C**) Correlation with immune checkpoint genes (inhibitory and stimulatory checkpoints). Spearman correlation coefficients are color-coded (red = positive, blue = negative). Asterisks denote statistical significance after FDR correction (* *p* < 0.05).

Given the traditional role of snoRNAs in rRNA modification, we investigated the correlation between SNORA12 and 44 genes involved in three major RNA modification pathways (m6A, m5C, m1A) across 19,131 samples from the TCGA, TARGET, and GTEx databases (Figure 3A).

Significant associations were observed with several m6A regulators. SNORA12 expression was positively correlated with the m6A methyltransferase METTL3(writer; R ≈ 0.45, *p* < 0.001) and the m6A reader YTHDF1(R ≈ 0.38, *p* = 0.003). Conversely, SNORA12 was negatively correlated with the m6A demethylase FTO(eraser; R ≈ −0.32, *p* = 0.01). Correlations were also observed with m5C regulators, such as NSUN2 (writer; R ≈ 0.28, *p* = 0.02), and m1A regulators, such as ALKBH1 (eraser; R ≈ −0.25, *p* = 0.04).

Hypothesis Based on Correlation:

Based on these findings, we propose the following hypotheses regarding SNORA12 function:

1. m6A pathway involvement: The positive correlations with METTL3 and YTHDF1 suggest that SNORA12 may promote the translation efficiency of oncogenic mRNAs by enhancing m6A modification [6]. The negative correlation with FTO implies that SNORA12 may maintain a pro-tumorigenic phenotype by inhibiting m6A demethylation [7].
2. **Cross-regulation of rRNA modification:** As a box H/ACA snoRNA, SNORA12 is predicted to guide rRNA pseudouridylation. It is plausible that SNORA12 may indirectly affect the recruitment of m6A reader proteins by altering ribosome structure or function [8].

These hypotheses are based solely on correlational evidence and require experimental validation through techniques such as MeRIP-seq, RIP-seq, and ribosome profiling in future studies.

### 3.5. Correlation Between SNORA12 and Immune Regulatory Genes

To further explore the immunological role of SNORA12, we analyzed its correlation with 150 marker genes spanning five immune pathways (chemokines, receptors, MHC, inhibitors, stimulators) (Figure 3B). SNORA12 expression showed significant positive correlations with the chemokine CXCL9 (R = 0.35, *p* < 0.01) and the co-stimulatory molecule CD28 (R = 0.20, *p* < 0.05), suggesting a potential role in T cell recruitment and activation. In contrast, SNORA12 was negatively correlated with CXCL10 (R = −0.28, *p* < 0.05) and the immune checkpoint PDCD1 (encoding PD-1; R = −0.15, *p* < 0.05).

Analysis of 60 immune checkpoint genes revealed that SNORA12 was negatively correlated with inhibitory checkpoints CTLA4 (R = −0.21, *p* < 0.01) and PDCD1 (R = −0.17, *p* < 0.05), while positively correlated with the stimulatory checkpoint CD276 (R = 0.25, *p* < 0.01) and ICOS (R = 0.19, *p* < 0.05) (Figure 3C). These complex correlations suggest that SNORA12 may fine-tune the immune checkpoint landscape in a context-dependent manner.

### 3.6. Correlation Between SNORA12 Expression and Clinical Pathological

**Figure 4:**
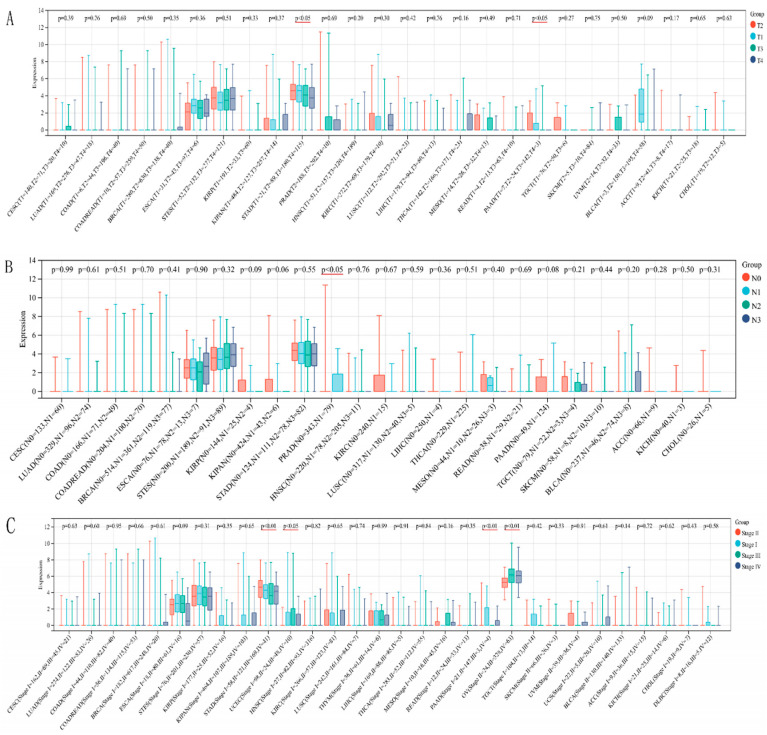
Correlation between SNORA12 expression and clinical pathological features across cancer types. Boxplots showing SNORA12 expression levels (log2(TPM+1) transformed) stratified by (**A**) T stage (primary tumor invasion depth), (**B**) N stage (lymph node metastasis status), and (**C**) clinical stage (AJCC clinical stage I–IV) for the indicated cancer types. Only cancer types with sufficient sample size in each subgroup are shown. Numbers in parentheses indicate sample sizes for each subgroup (e.g., T1, T2, T3, T4; N0, N1, N2, N3; Stage I, II, III, IV). *p*-values were calculated using the Kruskal–Wallis test (for ≥3 groups) or Wilcoxon rank-sum test (for 2 groups), and are displayed below each cancer type. Abbreviations: STAD, stomach adenocarcinoma; PAAD, pancreatic adenocarcinoma; HNSC, head and neck squamous cell carcinoma; UCEC, uterine corpus endometrial carcinoma; OV, ovarian serous cystadenocarcinoma; other abbreviations follow TCGA study abbreviations.

To investigate whether SNORA12 expression correlates with tumor progression and metastasis, we analyzed its relationship with three key clinical parameters, T stage (tumor invasion depth), N stage (lymph node metastasis status), and clinical stage (AJCC clinical stage I–IV), across multiple cancer types. Statistical comparisons were performed using the Kruskal–Wallis test (for multiple groups) or Wilcoxon rank-sum test (for two groups), and only cancer types with sufficient sample size in each subgroup were included. T stage (Figure 4A): Significant associations between SNORA12 expression and T stage were observed in two cancer types: stomach adenocarcinoma (STAD, N = 405, *p* = 0.03) and pancreatic adenocarcinoma (PAAD, N = 176, *p* = 0.04). In STAD, SNORA12 expression progressively increased from T1 to T3, suggesting a potential role in local tumor invasion. In PAAD, expression was significantly higher in T3 tumors compared to T1/T2. No significant correlations were found in the remaining 24 cancer types examined (e.g., LUAD, BRCA, COAD), indicating that the association with T stage is highly context-dependent. N stage (Figure 4B): Analysis of lymph node metastasis status revealed that only head and neck squamous cell carcinoma (HNSC, N = 514) exhibited a significant difference in SNORA12 expression across N0–N3 categories (*p* < 0.05). In HNSC, patients with lymph node metastasis (N1–N3) tended to have higher SNORA12 expression compared to node-negative patients (N0). No significant associations were detected in other cancer types (e.g., BRCA, LUAD, STAD, all *p* > 0.05), suggesting that SNORA12 is not broadly linked to nodal metastasis. Clinical stage (Figure 4C): When analyzing overall clinical stage (Stage I–IV), significant differences in SNORA12 expression were observed in four cancer types: stomach adenocarcinoma (STAD, N = 389, *p* < 0.01), uterine corpus endometrial carcinoma (UCEC, N = 180, *p* < 0.05), pancreatic adenocarcinoma (PAAD, N = 175, *p* < 0.01), and ovarian serous cystadenocarcinoma (OV, N = 415, *p* < 0.01). In these malignancies, SNORA12 expression tended to be higher in advanced stages (Stage III/IV) compared to early stages (Stage I/II), consistent with a potential oncogenic role in tumor progression. The remaining 26 cancer types showed no significant stage-related expression changes. Taken together, these results demonstrate that SNORA12 expression is associated with specific clinical features in a subset of cancer types, particularly STAD, PAAD, HNSC, UCEC, and OV, further supporting its context-dependent role in tumor biology.

### 3.7. SNORA12 Expression in Human Osteosarcoma Tissue

**Figure 5:**
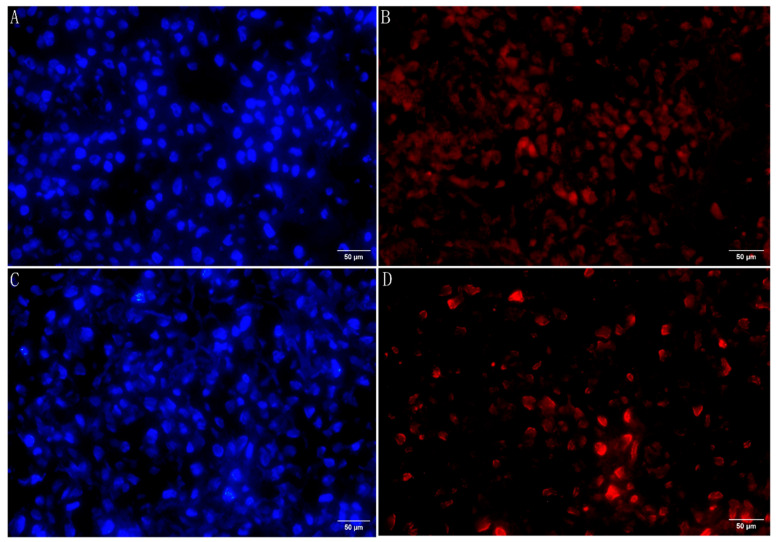
Fluorescent in situ hybridization (FISH) of SNORA12 in human osteosarcoma tissue. Representative images from two osteosarcoma patients: (**A**,**B**) 23-year-old male; (**C**,**D**) 56-year-old female. Red fluorescence: SNORA12 probe; blue fluorescence: DAPI nuclear stain. Scale bars = 50 μm.

To validate the clinical relevance of SNORA12 in sarcoma, we performed fluorescence in situ hybridization (FISH) on tissue sections from patients with osteosarcoma. As shown in Figure 5, SNORA12-specific red fluorescence signals were readily detectable in tumor cells from two representative patients (a 23-year-old male and a 56-year-old female)**.** DAPI counterstaining (blue) confirmed nuclear localization. These results confirm that SNORA12 is expressed in human osteosarcoma tissues, providing a rationale for functional studies on this malignancy.

### 3.8. SNORA12 Positively Regulates TIGIT Expression in Osteosarcoma Cells and NK Cells

Given the correlations observed between SNORA12 and immune checkpoint genes in our pan-cancer analysis (Figure 3C), we next investigated whether SNORA12 directly regulates TIGIT expression in osteosarcoma cells and NK cells.

First, we validated the efficiency of SNORA12 modulation by qRT-PCR. In SW1353 and U2OS osteosarcoma cells transfected with the SNORA12 overexpression plasmid (OE-SNORA12), SNORA12 levels were significantly increased compared to empty vector controls (OE-NC) (Supplementary Figure S2A,B). In NK92 cells transduced with shRNA targeting SNORA12 (sh-SNORA12), SNORA12 expression was significantly reduced compared to scrambled controls (sh-NC) (Supplementary Figure S2C). Additionally, successful overexpression of SNORA12 in primary human NK cells was confirmed (Supplementary Figure S2D).

**Figure 6:**
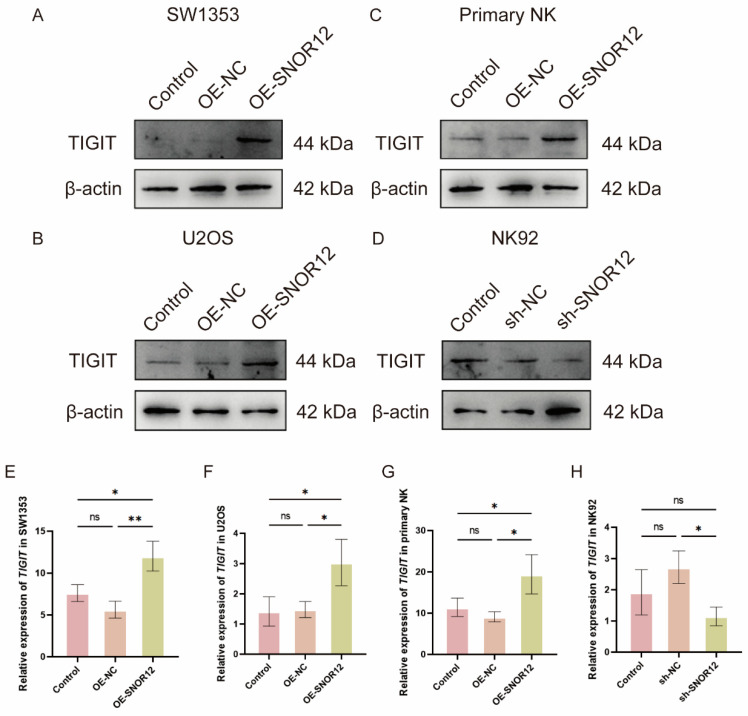
SNORA12 drives upregulation of the immune checkpoint TIGIT in osteosarcoma. (**A**–**D**) Representative Western blot analyses of TIGIT protein expression in the indicated cell lines under different treatments. SW1353 (**A**) and U2OS (**B**) osteosarcoma cells, as well as primary NK cells (**C**), were subjected to SNORA12 overexpression (OE-SNORA12), with respective controls (Control and OE-NC). NK92 cells (**D**), which endogenously express high levels of SNORA12, were subjected to SNORA12 knockdown (sh-SNORA12), with controls (Control and sh-NC). β-actin served as the loading control. (**E**–**H**) Quantitative analysis of TIGIT protein levels normalized to β-actin from (**A**–**D**), respectively. Data are presented as mean ± SD. Statistical significance was determined by Student’s *t*-test (* *p* < 0.05, ** *p* < 0.01, ns = not significant).

Overexpression of SNORA12 in SW1353 and U2OS cells led to significant upregulation of TIGIT at both the protein level (Figure 6A,B,E,F) and mRNA level. Similarly, overexpression of SNORA12 in primary human NK cells resulted in increased TIGIT protein expression (Figure 6C,G). Conversely, knockdown of SNORA12 in NK92 cells—which exhibit high endogenous SNORA12 expression—significantly reduced TIGIT protein levels (Figure 6D,H). Collectively, these results demonstrate that SNORA12 acts as a positive regulator of TIGIT in both osteosarcoma cells and NK cells, providing a potential mechanism by which SNORA12 may influence the immune landscape of the osteosarcoma microenvironment. However, whether SNORA12-mediated immune evasion is directly dependent on TIGIT upregulation requires further investigation through rescue experiments and in vivo models.

## 4. Discussion

This study presents a comprehensive pan-cancer analysis of SNORA12, revealing its tumor type-specific prognostic value, its correlation with immune infiltration and RNA modification pathways, and its previously unrecognized role as a positive regulator of the immune checkpoint TIGIT in osteosarcoma. These findings expand the current understanding of snoRNA functions beyond traditional ribosome biogenesis and position SNORA12 as a potential biomarker and therapeutic target.

### 4.1. Tumor Type-Specific Prognostic Value of SNORA12

A key finding of this study is the dual prognostic role of SNORA12: high expression was associated with poor prognosis in glioma but favorable outcomes in pancreatic and breast cancer. This “double-edged sword” effect likely reflects the heterogeneity of the tumor microenvironment (TME) and the context-dependent functions of snoRNAs. In metabolically active tumors such as pancreatic cancer, SNORA12 may exert a protective effect by maintaining ribosome homeostasis and protein synthesis fidelity through rRNA pseudouridylation [9]. In contrast, in highly invasive tumors like glioma, SNORA12 may promote stem-like properties and proliferation through non-canonical pathways, such as the PI3K-Akt signaling axis [10]. The lack of significant prognostic association in the majority of other cancer types further underscores that SNORA12 function is highly tissue- and context-dependent.

### 4.2. SNORA12 and the Immune Microenvironment: Correlations and Hypotheses

Our immune infiltration analysis revealed significant correlations between SNORA12 expression and multiple immune cell types across various cancers. Notably, the consistent positive association between SNORA12 and CD8+ T cell infiltration in THCA and LUAD across multiple algorithms (Supplementary Figure S1) suggests that SNORA12 may play a conserved role in promoting adaptive anti-tumor immunity in these cancer types. Conversely, the negative correlation with CD8+ T cells in glioma aligns with their poor prognostic value and supports a potential immunosuppressive role. The identification of seven cancer types (ACC, BRCA, COADREAD, ESCA, OV, READ, STES) with significant correlations across all six algorithms further highlights the broad immunological relevance of SNORA12, though the direction of these correlations varies and requires context-specific interpretation.

The correlation between SNORA12 and chemokine networks provides a potential mechanistic link. The positive correlation with CXCL9, a key chemokine for T cell recruitment [11], and the negative correlation with CXCL10, which can activate immunosuppressive signals under certain conditions [12], suggest that SNORA12 may shape the immune landscape by modulating chemokine expression. Additionally, the negative correlations with inhibitory immune checkpoints CTLA4 and PDCD1 raise the possibility that SNORA12 could enhance T cell activity by suppressing checkpoint expression [5]. However, these associations are correlational and require experimental validation.

### 4.3. SNORA12 and RNA Modification: A Basis for Mechanistic Hypotheses

The significant correlations observed between SNORA12 and key RNA modification regulators—particularly the m6A methyltransferase METTL3, the reader YTHDF1, and the demethylase FTO—suggest a potential interplay between snoRNA biology and epitranscriptomic regulation. Based on these findings, we propose the following hypotheses for future investigation:

1. **m6A-dependent oncogene regulation:** SNORA12 may promote the stability or translation efficiency of oncogenic mRNAs (e.g., MYC, EGFR) by enhancing m6A modification through positive regulation of METTL3 or inhibition of FTO [6,7].
2. **Ribosome-mediated indirect effects:** As a box H/ACA snoRNA, SNORA12 is predicted to guide rRNA pseudouridylation. Altered ribosome structure or function could indirectly affect the recruitment of m6A reader proteins to translating mRNAs [8].

These hypotheses are speculative and require rigorous experimental testing using techniques such as MeRIP-seq, polysome profiling, and site-directed mutagenesis of SNORA12.

### 4.4. SNORA12 as a Regulator of TIGIT: Implications and Limitations

The most significant mechanistic finding of this study is the demonstration that SNORA12 positively regulates TIGIT expression in both osteosarcoma cells and NK cells. TIGIT is an emerging immunotherapeutic target, and understanding the regulatory networks controlling its expression is of considerable clinical interest. Our data suggest that SNORA12 may contribute to an immunosuppressive microenvironment by upregulating TIGIT on NK cells and potentially tumor cells themselves.

However, several important limitations must be acknowledged:

1. Tumor-specific validation: The functional validation of SNORA12-mediated TIGIT regulation was performed exclusively in osteosarcoma models. Whether SNORA12 regulates TIGIT in other cancer types (e.g., glioma, pancreatic cancer) remains unknown and requires further investigation.
2. Lack of in vivo evidence: Our conclusions are based on in vitro cell line models. In vivo studies using xenograft or syngeneic mouse models are needed to determine whether SNORA12-driven TIGIT upregulation actually contributes to immune evasion and tumor growth.
3. Incomplete mechanistic depth: While we demonstrate that SNORA12 modulates TIGIT expression, the molecular mechanism—whether direct (e.g., through base-pairing interactions) or indirect (e.g., through RNA modification pathways)—remains undefined. Rescue experiments (e.g., overexpressing SNORA12 while blocking TIGIT) are required to establish functional dependence.
4. Bioinformatics reliance: The pan-cancer findings, while comprehensive, are primarily correlational and derived from public databases with inherent sample heterogeneity and batch effects.

### 4.5. Clinical Implications and Future Directions

Despite these limitations, our study has several potential clinical implications. First, the tumor type-specific prognostic value of SNORA12 suggests it could serve as a component of multi-gene prognostic panels for glioma, pancreatic cancer, and breast cancer. Second, the correlation with METTL3 and YTHDF1 raises the possibility that SNORA12 expression could predict response to therapies targeting the m6A pathway [13]. Third, the identification of SNORA12 as a positive regulator of TIGIT opens new avenues for combination immunotherapies—for example, targeting SNORA12 could potentially sensitize tumors to TIGIT blockade.

Future studies should focus on: (1) validating SNORA12 as a therapeutic target in in vivo osteosarcoma models; (2) investigating the SNORA12-TIGIT axis in other cancer types; (3) elucidating the molecular mechanism by which SNORA12 regulates TIGIT; and (4) exploring the clinical utility of SNORA12 as a predictive biomarker for immunotherapy response.

## 5. Conclusions

In summary, this pan-cancer analysis identifies SNORA12 as a tumor type-specific prognostic biomarker associated with immune infiltration and RNA modification pathways. Furthermore, we provide the first experimental evidence that SNORA12 positively regulates the immune checkpoint TIGIT in osteosarcoma cells and NK cells, suggesting a novel mechanism of snoRNA-mediated immune evasion. These findings warrant further investigation into the therapeutic potential of targeting the SNORA12-TIGIT axis in osteosarcoma and other malignancies.

## Figures and Tables

**Table 1:** Primer sequences used for qRT-PCR.

Primer	Sequence (5′ → 3′)
SNORA12 Forward	TTTTCAAATGGGCCTAACTCTGC
SNORA12 Reverse	AGATATCTCCGTCACAAGCCT
U6 Forward	TACGATACAAGGCTGTTAGAGA
U6 Reverse	ACTGCAAACTACCCAAGAAA

## Data Availability

The datasets generated and analyzed during the current study are available from the corresponding author on request. The original data presented in the study (TCGA, TARGET, and GTEx) are openly available in public repositories. The data can be accessed via the UCSC Xena portal (https://xenabrowser.net/) (accessed on 5 March 2026) and the official databases: TCGA (https://portal.gdc.cancer.gov/) (accessed on 5 March 2026), TARGET (https://ocg.cancer.gov/programs/target) (accessed on 5 March 2026), and GTEx (https://gtexportal.org/) (accessed on 5 March 2026).

## Supplementary Materials

The following supporting information can be downloaded at https://www.mdpi.com/article/10.3390/biomedicines14030723/s1: Figure S1: Intersection analysis of cancer types with significant SNORA12–CD8^+^T cell correlations across six algorithms.; Figure S2: Validation of SNORA12 overexpression and knockdown efficiency.
