# Supplementary figures and images for: Pan-Cancer Analysis Identifies SNORA12 as a Prognostic Biomarker and Demonstrates Its Role in Upregulating TIGIT in Osteosarcoma
Source: Biomedicines. 2026 Mar 20;14(3):723. doi: 10.3390/biomedicines14030723 (PMC13024225; doi:10.3390/biomedicines14030723)

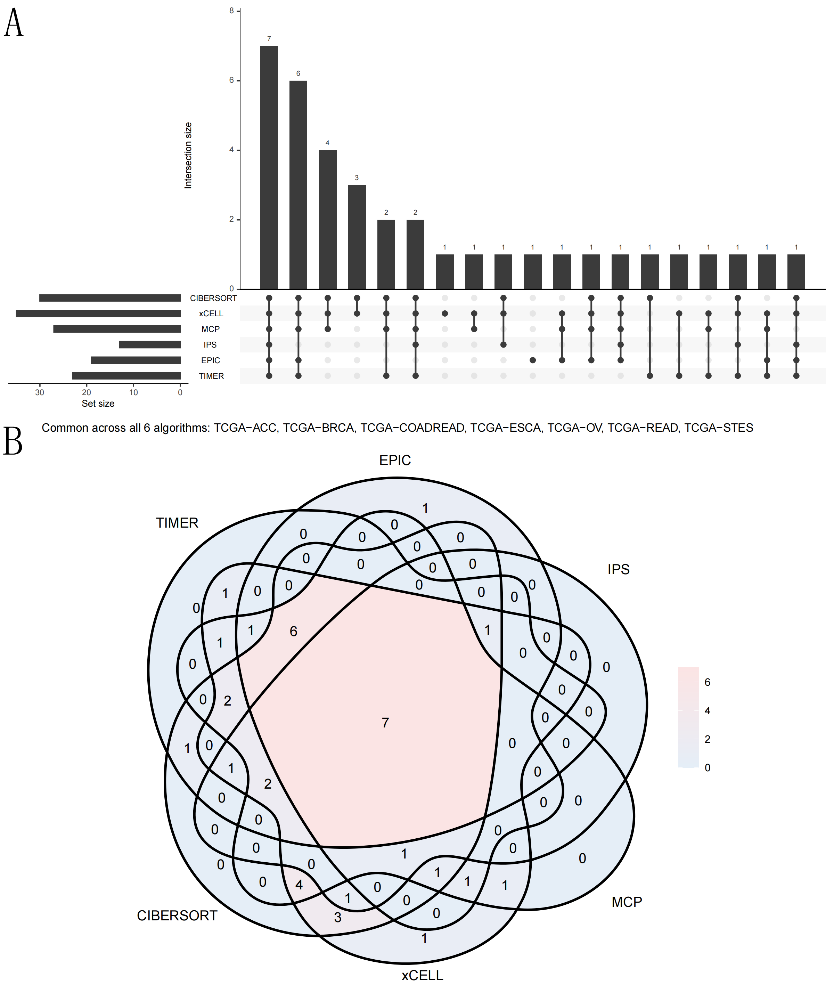

Supplement: Supplementary file 1 [file biomedicines-14-00723-s001.zip › Figure S1.png]

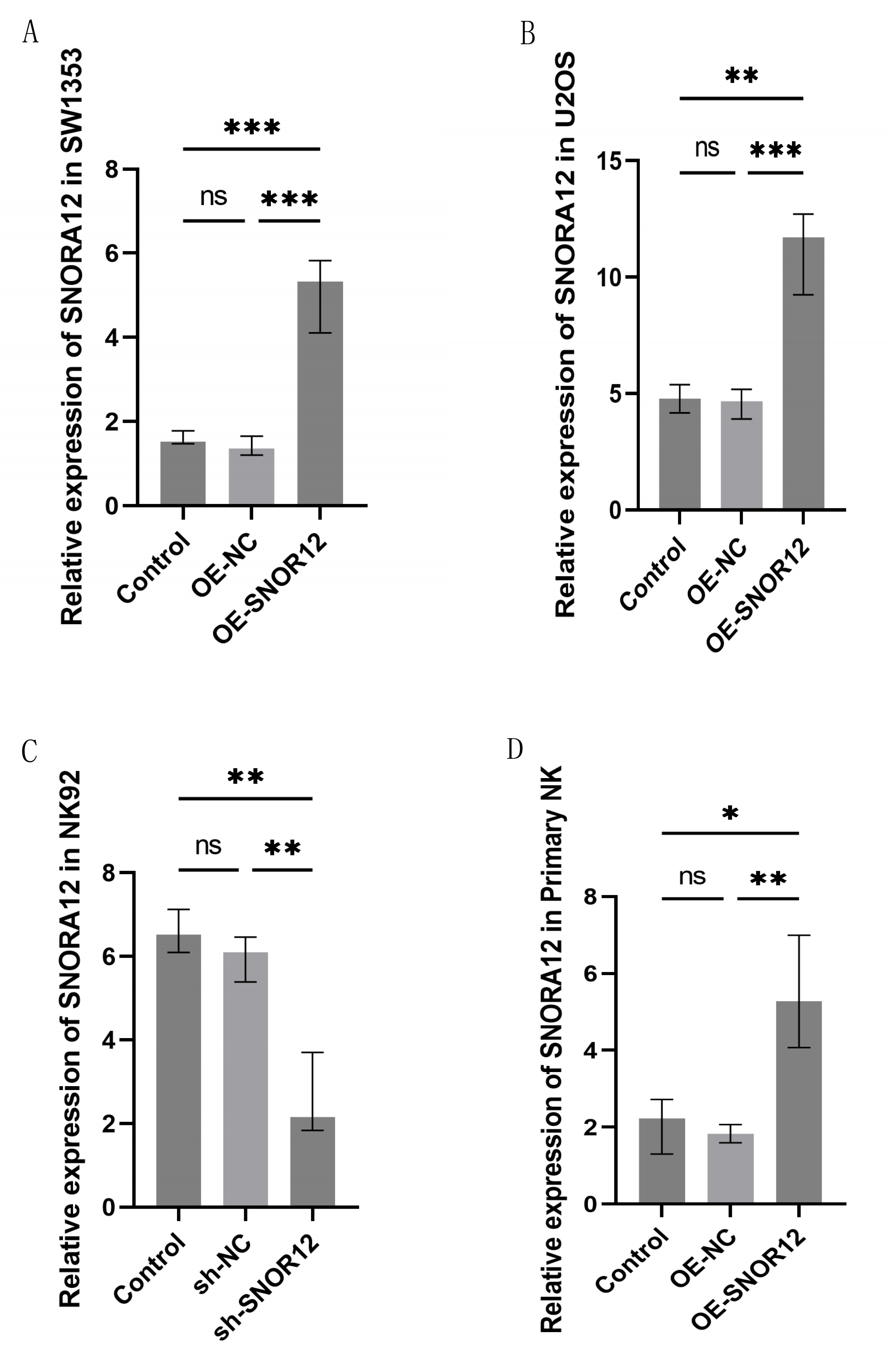

Supplement: Supplementary file 1 [file biomedicines-14-00723-s001.zip › Figure S2.png]
